# Simulation study for analysis of binary responses in the presence of extreme case problems

**DOI:** 10.1186/1297-9686-43-41

**Published:** 2011-11-30

**Authors:** Romdhane Rekaya, Robyn L Sapp, El H Hay, Ryan Davis, Joseph K Bertrand

**Affiliations:** 1 Animal and Dairy Science Department, University of Georgia, Athens 30602-2771, USA; 2 Department of Statistics, University of Georgia, Athens 30602-2771, USA; 3 Institute of Bioinformatics, University of Georgia, Athens 30602-2771, USA; 4 Cobb Vantress, Siloam Springs, Arkansas, USA 72761-1030

## Abstract

**Background:**

Estimates of variance components for binary responses in presence of extreme case problems tend to be biased due to an under-identified likelihood. The bias persists even when a normal prior is used for the fixed effects.

**Methods:**

A simulation study was carried out to investigate methods for the analysis of binary responses with extreme case problems. A linear mixed model that included a fixed effect and random effects of sire and residual on the liability scale was used to generate binary data. Five simulation scenarios were conducted based on varying percentages of extreme case problems, with true values of heritability equal to 0.07 and 0.17. Five replicates of each dataset were generated and analyzed with a generalized prior (**g-prior**) of varying weight.

**Results:**

Point estimates of sire variance using a normal prior were severely biased when the percentage of extreme case problems was greater than 30%. Depending on the percentage of extreme case problems, the sire variance was overestimated when a normal prior was used by 36 to 102% and 25 to 105% for a heritability of 0.17 and 0.07, respectively. When a g-prior was used, the bias was reduced and even eliminated, depending on the percentage of extreme case problems and the weight assigned to the g-prior. The lowest Pearson correlations between true and estimated fixed effects were obtained when a normal prior was used. When a 15% g-prior was used instead of a normal prior with a heritability equal to 0.17, Pearson correlations between true and fixed effects increased by 11, 20, 23, 27, and 60% for 5, 10, 20, 30 and 75% of extreme case problems, respectively. Conversely, Pearson correlations between true and estimated fixed effects were similar, within datasets of varying percentages of extreme case problems, when a 5, 10, or 15% g-prior was included. Therefore this indicates that a model with a g-prior provides a more adequate estimation of fixed effects.

**Conclusions:**

The results suggest that when analyzing binary data with extreme case problems, bias in the estimation of variance components could be eliminated, or at least significantly reduced by using a g-prior.

## Background

It is well known that when the binary responses (1 = cases and 0 = controls) associated with a particular level of an effect fall within the same category, being either all ones or all zeros (known as extreme case problems or **ECP**), the likelihood is under-identified [1] and variance components tend to be biased. Sorensen et al. [2] noted that with an increasing number of fixed effects for a constant number of observations, a larger proportion of fixed effect levels will contain ECP. The authors further noted that no information is present in the data to estimate these fixed effects and the likelihood is ill-conditioned. Moreno et al. [3] reported that marginal maximum likelihood yielded biased inferences about the variance component, and that the direction of the bias depended on the amount of information associated with either fixed or random effects. Furthermore, when the Gibbs sampler was used to perform the marginalization, positively biased inferences were reported when the amount of data per fixed effect was small [3]. The authors concluded that the bias persisted when fixed effects were poorly estimated, despite the large amount of information about the variance component.

Sorensen et al. [2] hypothesized that the choice of the prior distribution for the fixed effects could be the most critical element for dealing with ECP. Hoeschele and Tier [4] conducted a simulation study which indicated that a portion of the bias could be alleviated by assuming that fixed effects follow a priori a Gaussian distribution. Likewise, Moreno et al. [3] reported a reduction in the bias when a Gaussian probability density function was assigned to the prior distribution of the fixed effects. However, this strategy may not always work as Moreno et al. [3] reported that assigning a Gaussian probability density function to the prior distribution of the fixed effects did not reduce the bias for very sparse data structures. The objective of the current study was to investigate the effect on inferences of assigning a generalized prior (g-prior) with varying weights to fixed effects, first proposed by Zellner [5], for the analysis of binary data in the presence of varying percentages of ECP. A simulation using a sire model was used to test the effect of the g-prior in the presence of ECP.

## Methods

The threshold model is becoming a standard tool to analyze of discrete data in the field of animal breeding and genetics and extensive literature on its theoretical basis, implementation and application has been generated in the last twenty years [2,6,7].

Let *Y_ijk _*represent the observed binary trait taking values of 1 for cases or 0 for controls, and *l_ijk _*is an underlying continuous variable that relates to *Y_ijk _*through the following relationship:

$$Y_{ijk}=\left\{\begin{array}{c} 1\;if\;l_{ijk}>0\\ 0\;if\;l_{ijk}\leq 0 \end{array}\right)$$

Assuming the following mixed linear model on the liability scale:

(1) l=xβ+Zs+ee~N(0,I)

where ***l***, ***β ***and ***s ***are the vectors of liabilities, systematic and random effects, respectively. **I **is the identity matrix and **X **and **Z **are known incidence matrices.

To complete the Bayesian formulation, prior information for the model parameters must be specified. For the random effects, a multivariate normal distribution was assumed:

(2) $$p\left(s|A,\sigma_{0}^{2}\right)\sim N\left(0,A\sigma_{0}^{2}\right)$$

and a scaled inverted chi-squared density was assumed for $\sigma_{u}^{2}$

(3) $$p\left(\sigma_{u}^{2}|\nu_{u},\sigma_{u}^{2}\right)\sim \chi^{-2}\left(\nu_{u},\sigma_{u}^{2}\right)$$

with *ν_u _*and $\sigma_{0}^{2}$ set equal to 2 and the true value of the sire variance (0.05 or 0.02), respectively.

For the systematic effects, let $\beta ={\left[\beta_{ECP}^{\prime },\beta_{N\text{\_}ECP}^{\prime }\right]}^{\prime }$ where ***β***_*ECP *_and ***β***_*N_ECP *_are the ECP and non-ECP classes. For ***β***_*N_ECP*_, the following prior was assumed:

$$\beta_{N\text{\_}ECP}\sim N\left(0,I\sigma^{\text{2}}\right)\;\text{with}\;\sigma^{\text{2}}=10^{6}$$

For ***β***_*ECP*_, a generalized or g-prior was assumed. It consists of a special form of a natural conjugate prior distribution for the parameters of multiple regression models, and can be viewed as a reference informative prior [5]. Its advantage in dealing with the ECP is that it can restrict location parameters from taking extreme large values due to an ill-defined likelihood function. In its original form, a g-prior is defined as a normal distribution with a zero mean and a variance proportional to the standard errors of the least square (LS) estimators of the model parameters. However, a more generalized g-prior allows for a non-zero mean. The general form of a g-prior is given by:

$$\beta_{ECP}|\beta_{0},V\sim N\left(\beta_{0},V\right)$$

and

(4) $$V^{-1}=\frac{g}{\sigma_{e}^{2}}X_{ECP}^{\prime }X_{ECP}$$

where $\sigma_{e}^{2}$ is the residual variance and was set to one, ***β*_0 _**is the prior mean for the ECP classes, which could be set equal to the average of the non ECP classes, g is the relative weight given to the prior and ranges between 0 and 1, and ***X_ECP_***is the incidence matrix corresponding to the ECP classes.

Following Albert and Chib [8] and Sorensen et al. [2], all conditional posterior distributions are in closed form, normal for **β **and ***s***, truncated normal for ***l ***and scaled inverted Chi square for ?4?. Specifically, for ***β***_*ECP*_, the conditional distribution is:

(5) $$\begin{array}{c} p\left(\beta_{ECP}|\beta_{N\text{\_}ECP},g,\beta_{0},\sigma_{e}^{2},I\right)\\ \sim N\left(\frac{{\hat{\beta }}_{ECP}+g\beta_{0}}{1+g},\sigma_{e}^{2}{\left[\left(1+g\right)X_{ECP}^{\prime }X_{ECP}\right]}^{-1}\right) \end{array}$$

where ${\hat{\beta }}_{ECP}$ is the estimate of the effects of the ECP classes based on data and assuming that the $X_{ECP}^{\prime }X_{ECP}$ is full rank. The vector ***β***_0 _is, as indicated before, the mean of the normal prior for the ECP classes. Although it could be set to any reasonable value, including zero, in our case it was set equal to the average of the solutions for the non-ECP classes. Note that when g = 0, the conditional distribution in (5) reduces to that obtained when a flat prior is used.

## Simulation

A simulation using a sire model was carried out to investigate methods of analyzing binary data with ECP. Four over-lapping generations were simulated. The base population included 50 unrelated sires and subsequent generations consisted of 150 sires each. Thus, a total of 500 sires were generated. For animals in a given generation, their sires were selected at random from the pool of sires from all previous generations. The dataset consisted of 5000 daughter records from the 500 sires with an average of around ten records per sire. Classes of the fixed effects were randomly assigned to each record.

A linear mixed model which included a fixed effect and random effects of sire and residual was used to generate the binary responses. The fixed effect has 200 classes and was drawn from a uniform distribution with on average 25 observations per class. In order to obtain a desired percentage of ECP in the data, the bounds of the uniform distributions were adjusted. For example, by decreasing the lower bound of the uniform distribution (on the negative side of the real line), more liabilities will have negative values and thus their associate binary responses will be equal to zero which, in turn, will lead to more ECP classes. However, within a given percentage of ECP, only the seed for the random number generator was changed, not the bounds of the uniform distribution. The resulting incidence rate ranged from 9 to 34%, depending on the different simulation scenarios. Transmitting ability of individuals from the base population was sampled from a normal distribution, **$N\left(0,I\sigma_{u}^{2}\right)$**, where **I **was the identity matrix and $\sigma_{u}^{2}=0.05\;\text{or}\;0.02$. The remaining sire effects were sampled from a normal distribution with a mean equal to one half of the grandsire's transmitting ability and a variance equal to $\left(\frac{3}{4}\right)\sigma_{u}^{2}$. The residual term was sampled from a normal distribution, $N\left(0,I\sigma_{e}^{2}\right)$, where **I **was the identity matrix and $\sigma_{u}^{2}=1.0$, resulting in a heritability of 0.17 or 0.07.

For each daughter record, the liability was calculated as the sum of all effects included in the model. Binary responses were assigned so that if the liability was greater than zero, then *y_i _*= 1, otherwise *y_i _*= 0. For each one of the two values used for the sire variance, five datasets with a varying percentage of ECP were simulated: 5% (**5E**), 10% (**10E**), 20% (**20E**), 30% (**30E**), and 75% (**75E**). Five replicates were simulated for each level of ECP.

Four methods to account for ECP, using a standard threshold model, were investigated: normal prior (**NP**), 5% g-prior (**5G**), 10% g-prior (**10G**), and 15% g-prior (**15G**). All datasets were analyzed using the four methods to account for ECP; i.e., the 5% ECP data were analyzed using the NP, 5%, 10%, and 15% g-prior, which are denoted as 5E-NP, 5E-5G, 5E-10G, and 5E-15G, respectively. The remaining ECP datasets are similarly denoted with the percentage of ECP listed first and then the percentage of g-prior used in the analyses.

## Convergence

Convergence diagnostics were based on the method of Raftery and Lewis [9], as implemented in the CODA software [10]. The required burn-in period was always less than 1 300 iterations for all parameters in the analyses. Thus, a total chain length of 75 000 iterations of the Gibbs sampler was run with a conservative burn-in of 25 000 iterations. The remaining 50 000 iterations were retained without thinning for post-Gibbs analysis. Furthermore, point estimates of the mean and standard deviation, and the high posterior density 95% [**HPD (95%)**] interval were obtained from the CODA software [10] for all parameters.

## Results

Summaries of the posterior mean, standard deviation and HPD (95%) interval averaged over five replicates, are presented in Table 1 (when true sire variance was equal to 0.05) and Table 2 (when true sire variance was equal to 0.02). Using NP, point estimates of sire variance for 5E, 10E, and 20E, were greater than the true values but did not seem to be biased as the true value (0.05 or 0.02) was within the HPD (95%) interval. For 30E and 75E, the sire variance was grossly over-estimated and biased as the true value was outside or barely within the HPD (95%) interval. In general, the sire variances decreased with an increase in the percentage of g-prior used in the threshold analysis. When true sire variance was equal to 0.05, for 5E, the point estimate of the sire variance was 0.068 when NP was used; however, the point estimate decreased to 0.048 when 15G was used. This same trend was also observed for 10E (0.072 decreased to 0.050), 20E (0.082 decreased to 0.055), 30E (0.094 decreased to 0.058); and 75E (0.109 decreased to 0.069). Similar results were observed when the true value of the sire variance was 0.02 (Table 2). Analysis 15G yielded point estimates extremely similar to the true value for the 5E (0.048) and 10E (0.050) data. Furthermore, 15G yielded point estimates with values most similar to the true value and estimates with the smallest amount of bias for the 20E (0.055), 30E (0.058) and 75E (0.069) data.

**Table 1:** Posterior means (standard deviation) and the high posterior density 95% intervals of sire variance (true value = 0.05) for data with varying percentages of extreme case problems (ECP) (1,2)

ECP	NP^3^		5G		10G		15G	
5%	0.068 (0.018)		0.064 (0.017)		0.054 (0.015)		0.048 (0.014)	
10%	0.072 (0.021)		0.068 (0.018)		0.057 (0.016)		0.050 (0.015)	
20%	0.082 (0.026)		0.077 (0.022)		0.063 (0.020)		0.055 (0.018)	
30%	0.094 (0.033)		0.081 (0.026)		0.066 (0.023)		0.058 (0.021)	
75%	0.109 (0.039)		0.087 (0.037)		0.071 (0.032)		0.069 (0.028)	
								
	**High posterior 95% interval**							
	
	**NP**		**5G**		**10G**		**15G**	
	
	**HL**	**HU**	**HL**	**HU**	**HL**	**HU**	**HL**	**HU**

5%	0.038	0.103	0.033	0.098	0.026	0.085	0.021	0.077
10%	0.036	0.116	0.034	0.105	0.026	0.090	0.021	0.081
20%	0.041	0.132	0.036	0.122	0.026	0.103	0.021	0.093
30%	0.046	0.158	0.033	0.133	0.024	0.111	0.019	0.099
75%	0.056	0.210	0.045	0.168	0.036	0.157	0.029	0.122

^1^The values reported are the average of five replicates

^2^The true value of sire variance used in the simulation was 0.05

^3^NP = normal prior; 5G = 5% generalized prior; 10G = 10% generalized prior; and 15G = 15% generalized prior

^4^HL = lower bound of the high posterior density 95% interval; and HU = upper bound of the high posterior 95% interval

**Table 2:** Posterior means (standard deviation) and the high posterior density 95% intervals of sire variance (true value = 0.02) for data with varying percentages of extreme case problems (ECP) 1,2

ECP	NP^3^		5G		10G		15G	
5%	0.025 (0.014)		0.025 (0.014)		0.022 (0.009)		0.019 (0.009)	
10%	0.032 (0.015)		0.028 (0.013)		0.025 (0.011)		0.021 (0.010)	
20%	0.036 (0.017)		0.032 (0.015)		0.026 (0.012)		0.023 (0.011)	
30%	0.038 (0.017)		0.033 (0.016)		0.028 (0.014)		0.024 (0.011)	
75%	0.041 (0.029)		0.037 (0.018)		0.031 (0.015)		0.027 (0.012)	
								
	**High posterior 95% interval**							
	
	**NP**		**5G**		**10G**		**15G**	
	
	**HU **	**HL**	**HL HU**		**HL**	**HU**	**HL**	**HU**

5%	0.005	0.068	0.005	0.067	0.008	0.056	0.008	0.043
10%	0.012	0.077	0.009	0.068	0.010	0.053	0.011	0.045
20%	0.015	0.091	0.010	0.078	0.010	0.061	0.011	0.051
30%	0.018	0.103	0.014	0.084	0.012	0.064	0.012	0.057
75%	0.023	0.115	0.017	0.089	0.013	0.072	0.014	0.064

^1^The values reported are the average of five replicates

^2^The true value of sire variance used in the simulation was 0.02

^3^NP = normal prior; 5G = 5% generalized prior; 10G = 10% generalized prior; and 15G = 15% generalized prior

^4^HL = lower bound of the high posterior density 95% interval; and HU = upper bound of the high posterior 95% interval

A summary of the Pearson correlations between true and estimated fixed and random effects, averaged over five replicates, are presented in Table 3. For a heritability of 17%, Pearson correlations between true and estimated fixed effects for 5E (0.82), 10E (0.75), 20E (0.71), 30E (0.67), and 75E (0.49) were lowest when NP was used. The Pearson correlations between true and estimated fixed effects were virtually the same for 5E-5G (0.903), with 5E-10G (0.909) and 5E-15G (0.909). This same trend was also observed for the 10E, 20E, 30E, and 75E data. Similar results in magnitude and trend were observed when the heritability was equal to 7%. For the sire effect, Pearson correlations between true and predicted breeding values were virtually the same across g-prior weights within a given percentage of ECP.

**Table 3:** Pearson correlations between true (TR) and estimated effects for varying percentages of extreme case problems (ECP) for binary data with heritability of 0.17. (1)

	Fixed effect			
	
ECP	TR-NP^2^	TR-5G	TR-10G	TR-15G
5%	0.817	0.903	0.909	0.909
10%	0.754	0.896	0.901	0.902
20%	0.712	0.877	0.880	0.879
30%	0.669	0.859	0.859	0.856
75%	0.487	0.764	0.776	0.805
				
	**Sire effect**			
	
**ECP**	**TR-NP**	**TR-5G**	**TR-10G**	**TR-15G**

5%	0.534	0.534	0.534	0.534
10%	0.519	0.520	0.520	0.520
20%	0.465	0.465	0.465	0.465
30%	0.426	0.427	0.426	0.426
75%	0.378	0.378	0.379	0.379

^1^The values reported are the average of five replicates

^2^NP = normal prior; 5G = 5% generalized prior; 10G = 10% generalized prior; and 15G = 15% generalized prior

## Discussion

The results of this study indicate that point estimates of sire variance (Tables 1 and 2) obtained from 5E-NP, 10E-NP, and 20E-NP were slightly higher than the true values, although without any indication of noticeable bias. However, point estimates of sire variance obtained from 30E-NP and 75E-NP were severely biased. Depending on the percentage of ECP, biases ranged from 36 to 102% when true sire variance was equal to 0.05 and from 25 to 105% when true sire variance was equal to 0.02 when NP was used. In fact, the true value for sire variance was outside the HPD (95%) interval for 75E-NP, and barely inside the interval for 30E-NP for both heritability values.

When a g-prior was used, the bias was reduced and even eliminated, depending on the percentage of ECP and the weight assigned to the g-prior. With 5G, overestimation persisted for all five ECP datasets (Tables 1 and 2). Furthermore, the true value used in the simulation was closer to the lower bound of the HPD (95%) interval, especially for 30E-5G and 75E-5G, indicating that point estimates of sire variance are less likely to be similar to the true value. Thus, point estimates of sire variance are biased upward. In fact, point estimates of sire variance using both heritability values were biased upward by 25 to 85% when 5G was used. Conversely, when 10G was included in the analysis, the bias was eliminated for datasets 5E (0.054) and 10E (0.057) and significantly reduced for datasets 20E (0.063); 30E (0.066); and 75E (0.071). Using 15G, bias was completely eliminated, regardless of the percentage of ECP, and only a slight overestimation was observed for 75E-15G. The true value for sire variance was well centered within the HPD (95%) interval for 5E-15G, 10E-15G, 20E-15G, 30E-15G; and 75E-15G further indicating that a threshold model with a 15% g-prior resulted in more accurate estimation of sire variance for binary data with 5, 10, 20, 30, and 75% ECP.

The results of this study prove that when analyzing binary responses in the presence of ECP, a standard threshold model with a normal prior could lead to biased variance components, especially when the percentage of ECP is greater than 30%. Depending on the percentage of ECP in the dataset and on the weight assigned to the g-prior, bias in variance component estimation could be avoided. Although these results are promising, the univariate sire model is not the typical method to analyze a binary trait. Typically, binary traits are analyzed in combination with a continuous trait(s) using an animal model rather than a sire model. Thus, further testing is needed to apply this methodology to a more realistic scenario like the use of an animal model and joint analysis of binary and continuous traits. On the practical side, it is worth mentioning that the normal prior approach could be a viable alternative when the ECP rate is low (below 30%) as reported in previous studies [3,4]. Additionally, the results obtained using the normal prior could change if different hyper-parameters or model implementations are adopted.

When compared with NP, a substantial increase in the Pearson correlations between true and estimated fixed effects were observed, for all percentages of ECP data, when 5G, 10G or 15G was used in the analysis to account for ECP (Table 3). When a 15% g-prior was used instead of a normal prior, Pearson correlations between true and fixed effects increased by 11, 20, 23, 27, and 60% for 5E, 10E, 20E, 30E, and 75E, respectively. These results suggest that a standard threshold model with a normal prior was not adequate to estimate fixed effects, especially when 30% or more ECP were present. Conversely, when a g-prior was used, Pearson correlations between true and estimated fixed effects were virtually the same within datasets of varying percentages of ECP. This result suggests that a standard threshold model with a g-prior provides more adequate estimation of fixed effects.

There were no observable differences in Pearson correlations (Table 3) between true and predicted breeding values between using NP, 5G, 10G, and 15G within datasets of varying percentages of ECP. Unlike the correlations for the fixed effects, the increase in correlations between true and predicted breeding values when a 5% g-prior was used instead of no g-prior was less than 1% for 5E, 10E, 20E, 30E, and 75E. This result suggests that the presence of ECP in the fixed effects of binary responses and the weight of the g-prior assigned did not impact prediction of breeding values. However, the effect of ECP on breeding value prediction could be greater in situations where an animal has only one record, which would be the case in an animal model.

In this study, the weight of the g-prior was assumed known. A better approach is to estimate this parameter in the model. However, we believe that in practical situations, trying to estimate "g" could lead to the original problem of an ill-defined likelihood, unless a somewhat strongly informative prior is used.

## Conclusions

The results of this study prove that estimates of sire variance using a normal prior could be severely biased when a substantial percentage of ECP is present in binary data. Depending on the percentage of ECP present in the data, these upward biases of estimates ranged from 25 to 105% when a normal prior was used for the fixed effects. However, when a generalized prior was used, the bias was reduced and even eliminated, depending on the percentage of ECP and the weight assigned to the generalized prior. Pearson correlations between true and estimated fixed effects were lowest when a normal prior was used for all percentages of ECP. Furthermore, the correlations were similar when a generalized prior was used for all percentages of ECP present in the binary data. The results suggest that when analyzing binary data in the presence of ECP, bias in the estimation of variance components can be eliminated, or at least significantly reduced by using a generalized prior for the fixed effects.

## List of abbreviations used

**5E**: 5% extreme case problems; **5G**: 5% generalized prior; **10E**: 10% extreme case problems; **10G**: 10% generalized prior; **15G**: 15% generalized prior; **20E**: 20% extreme case problems; **30E**: 30% extreme case problems; **75E**: 75% extreme case problems; **ECP**: extreme case problems; **g-prior**: generalized prior; **HPD (95%) = **high posterior density 95% interval; **NP**: normal prior

## Competing interests

The authors declare that they have no competing interests.

## Authors' contributions

Co-authors RR and JKB designed and supervised all aspects of the study. RLS conducted the majority of the analyses. RD and EHH contributed to the drafting, results interpretation and paper formatting. All authors contributed to the drafting and editing of the manuscript. All authors read and approved the final manuscript.
